# Usefulness of three-dimensional printing of superior mesenteric vessels in right hemicolon cancer surgery

**DOI:** 10.1038/s41598-020-68578-y

**Published:** 2020-07-15

**Authors:** Yigang Chen, Linjie Bian, Hong Zhou, Danping Wu, Jie Xu, Chen Gu, Xinqi Fan, Zhequn Liu, Junyi Zou, Jiazeng Xia, Zekuan Xu

**Affiliations:** 1 Department of General Surgery, The Affiliated Wuxi No. 2 People’s Hospital of Nanjing Medical University, 68 Zhongshan Road, Wuxi, 214002 Jiangsu People’s Republic of China; 2 Department of Radiology, The Affiliated Wuxi No. 2 People’s Hospital of Nanjing Medical University, Wuxi, 214002 People’s Republic of China; 3 Operating Room, The Affiliated Wuxi No. 2 People’s Hospital of Nanjing Medical University, Wuxi, 214002 People’s Republic of China; 4 Harbin JunYang Technology Co., Ltd, Harbin, 150000 People’s Republic of China; 5 Department of General Surgery, First Affiliated Hospital of Nanjing Medical University, 140 Hanzhong Road, Nanjing, 210029 People’s Republic of China

**Keywords:** Colon cancer, Gastrointestinal models

## Abstract

The anatomy of the superior mesenteric vessels is complex, yet important, for right-sided colorectal surgery. The usefulness of three-dimensional (3D) printing of these vessels in right hemicolon cancer surgery has rarely been reported. In this prospective clinical study, 61 patients who received laparoscopic surgery for right hemicolon cancer were preoperatively randomized into 3 groups: 3D-printing (20 patients), 3D-image (19 patients), and control (22 patients) groups. Surgery duration, bleeding volume, and number of lymph node dissections were designed to be the primary end points, whereas postoperative complications, post-operative flatus recovery time, duration of hospitalization, patient satisfaction, and medical expenses were designed to be secondary end points. To reduce the influence of including different surgeons in the study, the surgical team was divided into 2 groups based on surgical experience. The duration of surgery for the 3D-printing and 3D-image groups was significantly reduced (138.4 ± 19.5 and 154.7 ± 25.9 min vs. 177.6 ± 24.4 min, *P* = 0.000 and *P* = 0.006), while the number of lymph node dissections for the these 2 groups was significantly increased (19.1 ± 3.8 and 17.6 ± 3.9 vs. 15.8 ± 3.0, *P* = 0.001 and *P* = 0.024) compared to the control group. Meanwhile, the bleeding volume for the 3D-printing group was significantly reduced compared to the control group (75.8 ± 30.4 mL vs. 120.9 ± 39.1 mL, *P* = 0.000). Moreover, patients in the 3D-printing group reported increased satisfaction in terms of effective communication compared to those in the 3D-image and control groups. Medical expenses decreased by 6.74% after the use of 3D-printing technology. Our results show that 3D-printing technology could reduce the duration of surgery and total bleeding volume and increase the number of lymph node dissections. 3D-printing technology may be more helpful for novice surgeons.

**Trial registration**: Chinese Clinical Trial Registry, ChiCTR1800017161. Registered on 15 July 2018.

## Introduction

With the introduction of the concept of complete mesocolic excision (CME), lymph node dissection in laparoscopic surgery has become more standardized, and the effect of surgical treatment on colon cancer has improved^1,2^. However, CME is a challenging procedure for the surgeon^3^. Indeed, CME surgery for right colon cancer requires clear dissection of the superior mesenteric artery and vein (SMA and SMV) and their branches^4–6^. Moreover, the Henle trunk and right colonic and ileocolic artery and vein present important anatomical differences^7–9^. This increases the difficulty of the surgery, and, consequently, the time required for a novice surgeon to learn the surgical technique. Therefore, we propose a new method to determine the course of the mesenteric vessels, and devise an appropriate surgical plan prior to the need for surgery.

Three-dimensional (3D) computed tomography (CT) angiography is useful for preoperative evaluation of the vascular anatomy of patients with gastric and colorectal cancers^10^. Recently, CT angiography, CT colonography, and image fusion technology have seen great progress and have been used in the preoperative evaluation of colon cancer^11–13^. These imaging techniques can provide information regarding the right half of the colon, including the vascular anatomy and variations, which can be helpful for CME surgery^14,15^. Therefore, the use of 3D-imaging data before surgery has drawn considerable attention.

A further development of image reconstruction technology is the 3D printing of vessels^16,17^. Medical 3D printing has been applied in many clinical fields and has yielded good results^18–20^. However, not many studies have focused on the 3D printing of the superior mesenteric vessels, which is urgently needed for the preoperative display of blood vessels. In this study, we compared the effects of a preoperative 3D-printed model and 3D reconstruction image on laparoscopic right hemicolon cancer surgery and assessed the usefulness of 3D printing of the superior mesenteric vessels for this type of surgery.

## Results

Based on the detailed inclusion and exclusion criteria described in the Methods section, a total of 61 patients were included in the current study, and no patients dropped out between the time of study enrollment and the actual surgery. In this study, the superior mesenteric vessels can be printed within 24 h after the CT data are submitted before surgery.

### Operation time

We calculated the duration of surgery for each group, from the beginning to the end of the surgery after anesthesia. The duration of surgery for the 3D-printing and 3D-image groups were 138.4 ± 19.5 and 154.7 ± 25.9 min, respectively, and significant differences were found (*P* = 0.035). Notably, the duration of surgery for the 3D-printing and 3D-image groups were significantly shorter than that for the control group (*P* = 0.000 and *P* = 0.006, Table 1). Indeed, the duration of surgery was reduced by 22.1% and 12.9% after the use of 3D-printing and 3D-image technology, respectively.

**Table 1:** Comparison of short-term surgical indicators of the 3D-printing group, 3D-image group, and control group.

Variable	3D-printing group	3D-image group	Control group
Duration of surgery (min)	138.4 ± 19.5**^#^	154.7 ± 25.9**	177.6 ± 24.4
Bleeding volume (mL)	75.8 ± 30.4**^#^	97.6 ± 31.5*	120.9 ± 39.1
Number of lymph node dissections	19.1 ± 3.8**	17.6 ± 3.9*	15.8 ± 3.0
Postoperative flatus recovery time (days)	2.9 ± 1.2	3.1 ± 1.3	2.8 ± 1.2
Duration of hospitalization (days)	8.9 ± 1.7	9.3 ± 2.5	9.2 ± 2.6
Rates of postoperative complications (%)	17.6	17.2	16.7

*P < 0.05, when 3D-printing group or 3D-image group was compared with control group.

**P < 0.01, when 3D-printing group or 3D-image group was compared with control group.

^#^*P* < 0.05, when 3D-printing group was compared with 3D-image group.

In terms of the 2 surgical teams, the duration of surgery for group A was 172.5 ± 23.7 min, which was longer than that for group B (146.8 ± 26.7 min, *P* = 0.000). Both group A and B for the 3D-printing group showed reduced duration of surgery. There were significant differences between group A and group B for the 3D-printing group regarding the duration of surgery (*P* = 0.004, Table 2). For group A, there were no significant differences between the 3D-printing and 3D-image groups. However, the duration of surgery for the 3D-printing and 3D-image groups was significantly reduced compared to the control group (*P* = 0.000 and *P* = 0.022). For group B, there were no significant differences between the 3D-printing and 3D-image groups. The duration of surgery for the 3D-printing and 3D-image groups was significantly reduced compared to the control group (*P* = 0.000 and *P* = 0.011).

**Table 2:** Comparison of duration of surgery, bleeding volume, and number of lymph node dissections between group A and group B.

Variable	Operative team	Overall	3D-printing group	3D-image group	Control group
Duration of surgery (min)	Group A	172.5 ± 23.7**	152.5 ± 21.2**^##^	171.4 ± 21.5**^#^	191.4 ± 10.4*
	Group B	146.8 ± 26.7	128.2 ± 10.1^##^	139.7 ± 20.1^#^	168.0 ± 26.9
Bleeding volume (mL)	Group A	109.0 ± 32.1*	83.1 ± 16.2^##^ +	112.2 ± 33.8*	128.9 ± 26.7**
	Group B	83.7 ± 30.5	70.5 ± 37.4	84.5 ± 23.7	94.2 ± 26.0
Number of lymph node dissections	Group A	15.2 ± 3.5	17.4 ± 2.6	14.5 ± 3.2*	14.6 ± 3.8
	Group B	17.7 ± 4.2	20.7 ± 4.1^##^	19.0 ± 3.3^##^	15.5 ± 3.7

Group A. Surgeons with less surgical experience or novice surgeons.

Group B. Surgeons with more surgical experience.

*P < 0.05, when group A was compared with group B.

**P < 0.01, when group A was compared with group B.

^#^*P* < 0.05, when 3D-printing group or 3D-image group was compared with control group,

^##^*P* < 0.01, when 3D-printing group or 3D-image group was compared with control group.

^+^*P* < 0.05, when 3D-printing group was compared with 3D-image group.

### Bleeding volume

The total bleeding volumes for the 3D-printing and 3D-image groups were 75.8 ± 30.4 and 97.6 ± 31.5 mL, respectively, and significant differences were found between the 3 study groups (*P* = 0.036) (Table 1). The bleeding volume for the 3D-printing group was significantly lower than that of the control group (*P* = 0.000), and the bleeding volume for the 3D-image group was also lower than that of the control group (*P* = 0.045). The bleeding volumes were reduced by 37.3% and 19.3% after the use of 3D-printing and 3D-image technology, respectively.

In terms of the surgical teams, the bleeding volume for group A was 109.0 ± 32.1 mL, which was greater than that for group B (83.7 ± 30.5 mL, *P* = 0.003). There were no significant differences between group A and group B for the 3D-printing group regarding the bleeding volume (*P* = 0.358, Table 2). For group A, the bleeding volume for the 3D-printing group was significantly reduced compared to the control group (*P* = 0.001) and was also reduced compared to the 3D-image group (*P* = 0.043). For group B, there were no significant differences between the 3 groups.

### Number of lymph node dissections

The number of lymph node dissections for the 3D-printing and 3D-image groups was 19.1 ± 3.8 and 17.6 ± 3.9, respectively, and no significant differences were found (Table 1). The number of lymph node dissections for the 3D-printing and 3D-image groups was significantly increased compared to the control group (*P* = 0.001 and *P* = 0.024). The number of lymph node dissections increased by 20.9% and 11.4% after the use of 3D-printing and 3D-image technology, respectively.

In terms of the surgical teams, the number of lymph node dissections for group A was 15.2 ± 3.5, which was less than that for group B (17.7 ± 4.2 mL, *P* = 0.006). The number of lymph node dissections for the 3D-printing group in group A was 17.4 ± 2.6, which was similar to the number of dissections for the 3D-printing group in group B (20.7 ± 4.1, *P* = 0.069). For group A, the number of lymph node dissections for the 3D-printing and 3D-image groups was similar to that of the control group (Table 2). For group B, there was no significant difference in the number of lymph node dissections between the 3D-printing and 3D-image groups. The number of lymph node dissections for the 3D-printing and 3D-image groups was greater than that of the control group (*P* = 0.002 and 0.003).

### Postoperative flatus recovery time and hospital stay time

There were no significant differences in the postoperative flatus recovery time (2.9 ± 1.2, 3.1 ± 1.3, and 2.8 ± 1.2 days) and duration of hospitalization (8.9 ± 1.7, 9.3 ± 2.5, and 9.2 ± 2.6 days) between the 3D-printing, 3D-image, and control groups, respectively. All the patients started liquid food intake approximately 3 days after the surgery.

### Post-operative complications

In 61 patients who underwent CME surgery, 14 postoperative complications were detected, including 5 cases of lymphatic fistula (1 in the 3D-printing group, 2 in the 3D-image group, and 2 in the control group), 4 cases of incision fat liquefaction with infection (2 in the 3D-printing group, 1 in the 3D-image group, and 1 in the control group), 3 cases of intestinal obstruction (1 in the 3D-image group and 2 in the control group), and 2 cases of pulmonary infection (1 each in the 3D-image and control groups). The rates of post-operative complications were 17.6%, 17.2%, and 16.7% for the 3D-printing, 3D-image, and control groups, respectively. There were no significant differences between these groups.

### Patient satisfaction

An adapted, validated, and pre-tested patient satisfaction questionnaire (PSQ-18) was interviewer-administered to consenting patients. Patients in the 3D-printing group reported increased satisfaction than those in the 3D-image and control groups in terms of effective communication (Table 3).

**Table 3:** Patients’ satisfaction with 3D printing group, 3D image group, and control group.

Satisfaction domains	3D printing group (%)	3D image group (%)	Control group (%)
General satisfaction	17 (85.0)	14 (73.7)	16 (72.7)
Time spent with doctor	18 (90.0)	13 (68.4)	15 (68.2)
Manner of approach	16 (80.0)	15 (78.9)	18 (81.8)
Effective communication	19 (95.0)*^#^	13 (68.4)	14 (63.6)
Cost of services received	16 (80.0)	12 (63.2)	15 (68.2)
Accessibility and convenience	15 (75.0)	14 (73.7)	16 (72.7)

*P = 0.031, when 3D printing group was compared with 3D image group.

^#^*P* = 0.013 when 3D printing group was compared with No 3D group.

### Medical expenses

The medical expenses for the 3D-printing, 3D-image, and control groups were 38,926.4 ± 2,831.6, 40,089.9 ± 2,612.3, and 41,555.1 ± 3,390.1 RMB, respectively (approximately 5,744.3 ± 417.9, 5,916.0 ± 385.5, and 6,132.2 ± 500.3 USD, respectively). The medical expense for the 3D-printing group was significantly less than that of the control group (*P* = 0.008). The medical expense decreased by 6.74% after the use of 3D-printing technology. There were no differences between the 3D-printing and 3D-image groups and between the 3D-image and control groups. Other high medical expenses were further analysed as follows. There was no significant difference in the cost of both the surgery and anesthesia among the 3 groups, which were 13,192.3 ± 2,136.2, 12,998.7 ± 2,655.9, and 13,041.3 ± 2084.5 RMB respectively. The cost of nutrition support (including enteral nutrition and parenteral nutrition) for the 3D-printing, 3D-image, and control groups were 9,001.1 ± 2056.3, 10,792.3 ± 3,397.7, 12,138.2 ± 3,385.6 RMB respectively. The medical expense of nutrition support for the 3D-printing group was significantly less compared to the control group (*P* = 0.001). There was no significant difference in various medical examinations and tests among the 3 groups, which were 10,077.8 ± 1,187.4, 9,941.5 ± 956.3.9, and 9,580.1 ± 1,084.5 RMB respectively.

In this study, the 3D-printed model was funded by a project of the Jiangsu Provincial Health and Family Planning Commission for young talent’s subsidy in science and education (QNRC2016146) and the Wuxi Science and Technology Development Fund (CSE1N1707). The cost of 3D printing materials of each 3D-printed model was 2000 RMB (approximately 290.5 USD). However, some potential costs, such as the purchase cost of the 3D printer and the time dedicated by the professional for reconstructions and printing itself, make a 3D printing project an expensive project.

## Discussion

The anatomy of the superior mesenteric vessels is an important aspect of right-sided colorectal surgeries^10^. With the help of new surgical instruments and anastomotic materials, rapid improvements have been made in right colon cancer surgery. Currently, however, it is difficult to achieve major breakthroughs in surgical techniques and perioperative management. Many studies have focused on methods that use digital technology to improve the visualization of the mesenteric vessels before surgery to assist the surgery. However, the usefulness of 3D printing of the superior mesenteric vessels in right hemicolon cancer surgery is unclear. This study showed that for right colon cancer surgery, 3D printing presents an effective method that reduces the risk of surgery and duration of surgery, increases the therapeutic effect, and improves patient satisfaction. This method is particularly helpful for novice surgeons. For patients preparing for right-sided colon cancer surgery and for surgeons, especially novice surgeons, the preoperative 3D printing of mesenteric vessels is a good alternative.

The minimal 3D printing time before surgery provides sufficient time for the surgeon to refer to it before the surgery. Doctors can take the model in their hands before the surgery and see the mutation of the blood vessels and the interaction between tumours and blood vessels from different angles. Moreover, by placing 3D vascular models next to laparoscopic displays, surgeons can obtain real-time data if they encounter problems, such as vascular variability, during surgery. In this scenario, the operator is like a driver, and the variation in the 3D vascular model is similar to car navigation.

Mari et al. reported that preoperative 3D-CT angiography can significantly reduce the duration of surgery and the incidence of complications related to the difficult or erroneous identification of the mesenteric vessels^21^. Does 3D-printing technology based on 3D-CT angiography have obvious advantages over 3D-CT angiography? The results of this study showed that both 3D printing and 3D imaging are good for surgery, but the effect of 3D printing is more apparent. Indeed, 3D printing can shorten the duration of surgery independent of the experience of the surgeon. The pre-operative 3D printing of the superior mesenteric vessels can reduce the duration of surgery and bleeding volume to a greater extent than 3D imaging and can increase patient satisfaction. Particularly, for surgeons who have less experience, 3D-printing technology is more helpful in reducing bleeding than 3D-imaging-based 3D-CT angiography.

The experience of the surgeon is very important for right colon cancer surgery. The learning curve of CME surgery for colorectal cancer is approximately 25 cases^22,23^. Typically, surgeons with little surgical experience often require prolonged surgical time, cause more bleeding, and perform fewer lymph node resections. However, our study showed that with the help of the 3D-printing technology, novice surgeons had a similar amount of bleeding and lymph node resection compared to more experienced surgeons. These results suggested that 3D printing was more advantageous for surgeons with less experience in CME surgery.

The application of 3D printing in medical treatment is becoming a major area of focus. Currently, 3D-printing technology has been employed in clinical trials for vascular interventions, anatomical resection of hepatocellular carcinoma, and the treatment of intracranial aneurysms^24–26^. However, there have been very few reports of the use of 3D printing in colon surgery. In this study, the size ratio of the 3D-printed mesenteric vessel to the patient's true blood vessel was 1:1. The surgeon can tilt and rotate the 3D model at any angle before the surgery and observe all aspects of the lesion and/or blood vessels, among other aspects. These advantages aid in the development of the best surgical protocols to reduce the risk of vascular variability. Particularly for surgeons who have less experience performing surgeries that involve complicated blood vessels, detailed preoperative planning is important. However, our study investigated only a few short-term surgical indicators; long-term data still needs to be collected.

The 3D-CT angiography results are presented in the form of an image on a 2D screen. In contrast, the 3D-printed models can be physically held in one’s hand, which allows for a more intuitive observation. This is more in line with people's natural observation habits. In addition to the surgical benefits, we found that when a surgeon uses a 3D-printed model to explain the surgical plan to the patient, the patient is able to understand the surgical program and the risk of surgery more quickly and clearly. This enhances their confidence in the surgery and helps in establishing good communication channels between doctors and patients, as well as in facilitating postoperative recovery.

In this study, results showed that 3D printing technology could help reduce the cost of right hemicolon cancer surgery. The main reason consists in the reduced cost of postoperative nutrition, since reduced duration of surgery and less bleeding are associated with increased recovery after surgery. It should be noted that this study was performed in China, where the cost of design and manufacturing is low, so the understanding of cost calculation will be different from that in other parts of the world. Indeed, in other parts of the world, 3D printing technology may not have the same effect on reducing medical costs as reported in this study. Owing to the advancement and popularisation of 3D-printing technology, its cost has dropped significantly in recent years^27,28^. In this study, the 3D-printed model was funded by the project of the Jiangsu Provincial Health and Family Planning Commission for young talent’s subsidy in science and education (QNRC2016146) and Wuxi Science and Technology Development Fund (CSE1N1707), and the patients did not have to pay for the models. As the cost of 3D printing decreases and the number of cases increases, the medical expenses of patients will continue to decrease, which will greatly benefit the popularisation of this technology and as well as patients with right colon cancer.

## Conclusion

The usefulness of 3D printing of the superior mesenteric vessels in right hemicolon cancer surgery has rarely been reported in the literature. The results of this randomized prospective clinical study showed that 3D printing of superior mesenteric vessels can benefit laparoscopic CME surgery for right hemicolon cancer. We found that mesenteric vascular 3D-printing technology is more valuable for less-experienced surgeons. Our research has made a prospective attempt to apply 3D-printing technology to celiac vessels. In the future, 3D-printing technology can be applied to all surgeries requiring the dissection of blood vessels and lymph nodes (such as gastric and rectal cancer cases), which will ultimately benefit more patients.

## Methods

### Study design

This prospective, randomized controlled trial involved 61 consecutive patients who were admitted to the Affiliated Wuxi No. 2 People’s Hospital of Nanjing Medical University to undergo laparoscopic CME for right hemicolon cancer from July 1, 2018 to February 1, 2019. Informed consent was obtained from all the patients prior to participation, and the study was approved by the Ethics Committee of the Affiliated Wuxi No. 2 People’s Hospital of Nanjing Medical University. This study has been registered with the China Clinical Trial Registry (registration number: ChiCTR1800017161). All experiments were performed in accordance with relevant guidelines and regulations.

The exclusion criteria included: patients experiencing metastasis; with severe heart, lung, liver, or kidney disease; pregnant women; patients suffering from severe obesity (BMI ≥ 30) or C-TNM stage IV cancer; having a tumour with a diameter larger than 7 cm; or requiring emergency surgery for acute intestinal obstruction. The inclusion criteria were as follows: patients (1) who had carcinoma of the hepatic flexure, ascending colon, or cecum identified by pre-operative histopathological findings; (2) whose preoperative C-TNM stage was I, II, or III according to the American Joint Committee on Cancer criteria; and (3) whose tumour diameter was less than 7 cm. In 72 patients considered for the study, 4 were excluded because they did not agree to the terms stipulated in the consent form and 7 were excluded due to severe obesity, liver metastasis, or presenting a tumour diameter larger than 7 cm. Eventually, 61 patients were included in the study.

**Figure 1:**
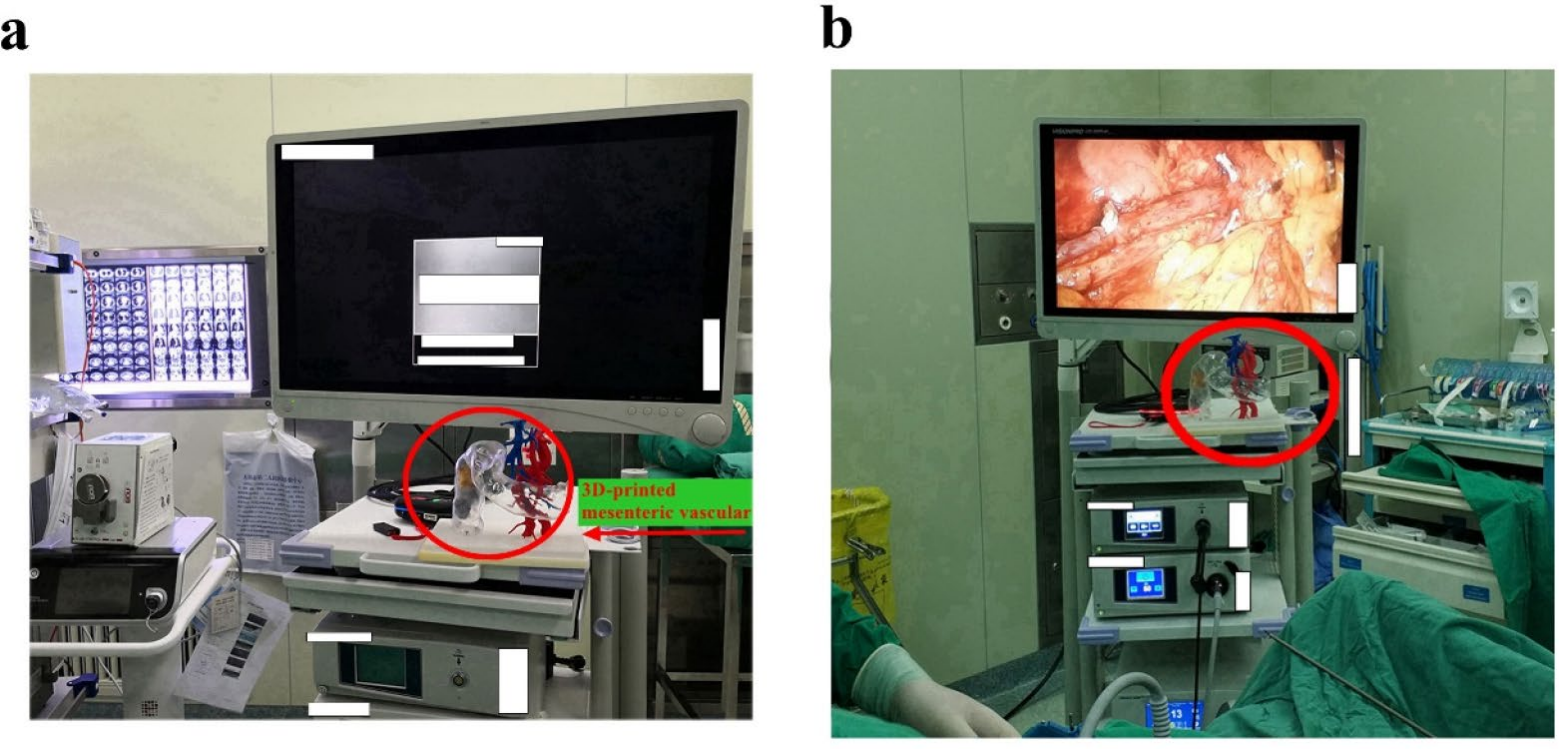
3D-printed mesenteric vascular model placed next to the monitor for reference during surgery. (**a**) Before surgery, 3D printed mesenteric vessels were placed under the monitor, (**b**) During operation, 3D printing mesenteric vascular model can provide direct reference for surgeons.

Patients were randomised into 3 groups: 3D-printing, 3D-image, and control groups. In the 3D-printing group, the superior mesenteric vessels of the patients were 3D printed and preoperatively reviewed by a surgeon. During surgery, a 3D-printed mesenteric vascular model was placed next to the monitor for reference (Fig. 1a). In the 3D-image group, patients were enrolled for preoperative 3D-image reconstruction by CT, and 3D printing was not performed for these patients. In the control group, patients were enrolled for only CT scan, and 3D-image reconstruction and 3D printing were not performed for these patients. The patient characteristics included age, gender, tumour size, tumour location, differentiation level, and tumour pathological stage (Table 4).

**Table 4:** Baseline patient characteristics.

Variable		N	3D-printing group	3D-image group	Control group	*P* value
Age		61	60.8 ± 18.3	65.4 ± 15.5	58.9 ± 17.9	0.85
Sex	Male	29	11	9	9	0.66
	Female	32	9	10	13	
Tumour size (cm)		61	3.92 ± 1.52	3.82 ± 1.58	3.29 ± 1.54	0.381
Tumour location	Cecum	5	1	2	2	0.308
	Ascending colon	29	9	9	11	
	Hepatic flexure	18	6	5	7	
	Transverse colon	9	4	3	2	
T stage	T1/2	33	11	9	13	0.754
	T3	28	9	10	9	
N stage	N0	21	7	6	8	0.306
	N1–3	27	9	8	10	
	N3–6	13	4	5	4	
Differentiation level	Well/moderate	33	12	11	10	0.538
	Poor/undifferentiated	28	8	8	12	
TNM stage	I/II	21	7	6	8	0.823
	III	40	13	13	14	

This study is a single-blind study, in which the surgeons were not blinded to the treatment group. After the selected patients provided informed consent, each patient was asked to select 1 of 3 opaque envelopes to be randomly assigned to a group. Patients who drew an envelope containing the letter P were assigned to the 3D-printing group; those who drew the letter I were assigned to the 3D-image group; and those who drew the letter C were assigned to the control group. Eventually, 20, 19, and 22 patients were assigned to the 3D-printing, 3D-image, and control groups, respectively. Surgery duration, bleeding volume, and number of lymph node dissections were designed to be the primary end points, whereas postoperative complications, the postoperative flatus recovery time, the duration of hospitalization, patient satisfaction, and medical expenses were designed to be secondary end points. To ensure the quality of the study and to reduce bias, only surgeons with experience in performing at least 10 laparoscopic colon cancer surgeries were selected to be included in the study.

### 3D-CT angiography

The 3D-CT angiography was performed on patients using a 320-slice CT scanner (Toshiba Aquilion ONE, Toshiba Co., Ltd., Japan). The patient was asked to breathe after inhaling deeply, and scanning was performed from the top of the diaphragm to the lower margin of the pubic symphysis. The contrast agent used was iohexol (BeiLu Pharmaceutical Co., Ltd., 320 mgI/mL, total volume: 90–100 mL, injection flow rate: 3 mL/s), which was injected using a high-pressure syringe via the elbow vein group. The time for the arterial phase scan after injection was 22–30 s, whereas the time for the portal venous scan was 60–72 s. The scanning parameters were as follows: tube voltage: 120 kV; automatic tube current (89–420 mAs); collimator width: 0.5 mm × 64 or 1 mm × 32; scan field: 30–35 cm; and ball tube speed: 0.5 s/w.

The image volume data were transmitted to a post-processing workstation (Advantage Workstation version 4.6, GE Medical Systems, USA). Arterial and venous revascularization and image fusion were performed. The blood vessels were reconstructed using the volume rendering method by 2 experienced radiologists. The original images of the arterial phase were combined with the images obtained using multi-planar reconstruction. Volume rendering reconstruction was carried out, and red pseudo-color was added. The vein of the intravenous phase was rebuilt using the same method, and blue pseudo-color was added. CT coronal imaging was performed on the coronal images of the venous phase. Virtual-reality images were used to create a 3D fusion image. All the images were interpreted by 2 surgeons (YC and JX) and 2 radiologists (LB and DW).

### Manufacturing of 3D-printed model

**Figure 2:**
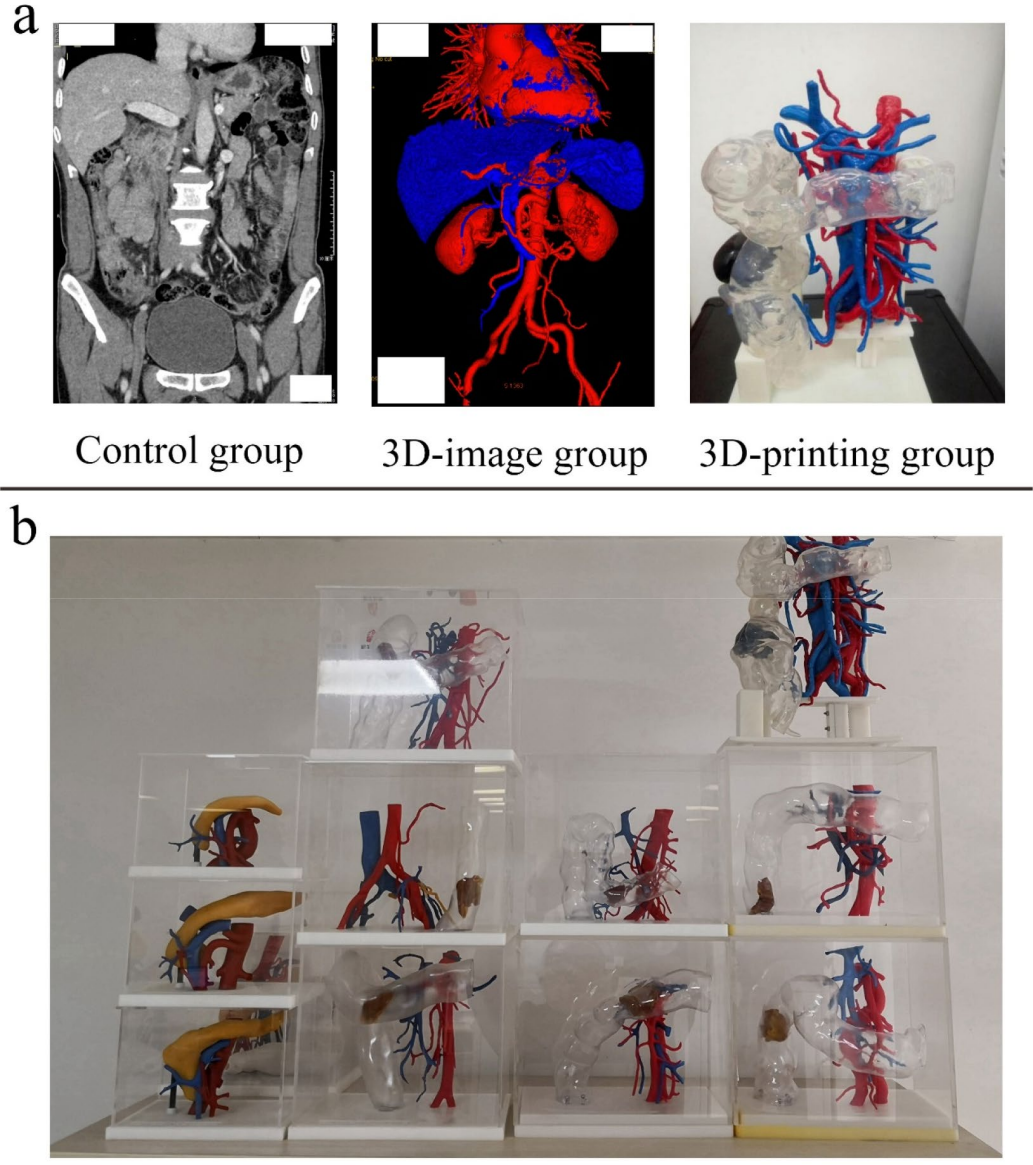
The sample images of the control group (2D CT data), 3D-image group, and 3D-printing group for the same patient (**a**), and the printed mesenteric blood vessels (including detachable right halves and tumours) (**b**).

The image data were collected and stored in the DICOM format. The 3D reconstruction was performed using software mimics 21 (Materialise, Belgium), and post processing software using Geomagic studio2014 (3D System, America) and ZBrush R8 (Pixologic, America). The information was pre-processed to remove the data of the bone and viscera and to retain the vessels surrounding the superior mesenteric vessels. Based on the pre-treated model files, the smoothing model was further optimized, and the supporting base parts were designed. To ensure good strength and toughness, the blood vessels were printed using thermoplastic urethane. Since we intended for the tumour in the intestine to be represented in the model, the right hemicolon was printed using a highly transparent resin, and the tumour was simulated using silica gel. The base of the 3D model was printed using resin material. The examples of 3D printing, 3D reconstruction images, and CT scan images are shown in Fig. 2a, and the printed mesenteric blood vessels (including detachable right halves and tumours) are shown in Fig. 2b.

### Operation processes

All surgeons involved in this study have participated in laparoscopic surgery training and obtained national recognized qualifications and certificates. Before the surgery, the 3D-printed model of the superior mesenteric vessels (3D-printing group), 3D reconstruction image (3D-image group), or two-dimensional (2D) CT scan image (control group) was observed by the surgeons. To reduce the influence of including different surgeons in the study, the surgical team was divided into 2 groups based on surgical experience, more specifically, number of previous right colon cancer operations: 10–25 cases (group A) and greater than 25 cases (group B). The patient was told that they would be randomly divided into group A and group B, understood the difference between group A and group B, and signed the informed consent form. In this study, group A completed 26 cases of CME surgery (8 cases of the 3D-printing group, 9 cases of the 3D-image group, and 9 cases of the control group). Meanwhile, group B completed 35 cases of CME surgery (12 cases of the 3D-printing group, 10 cases of the 3D-image group, and 13 cases of the control group).

**Figure 3:**
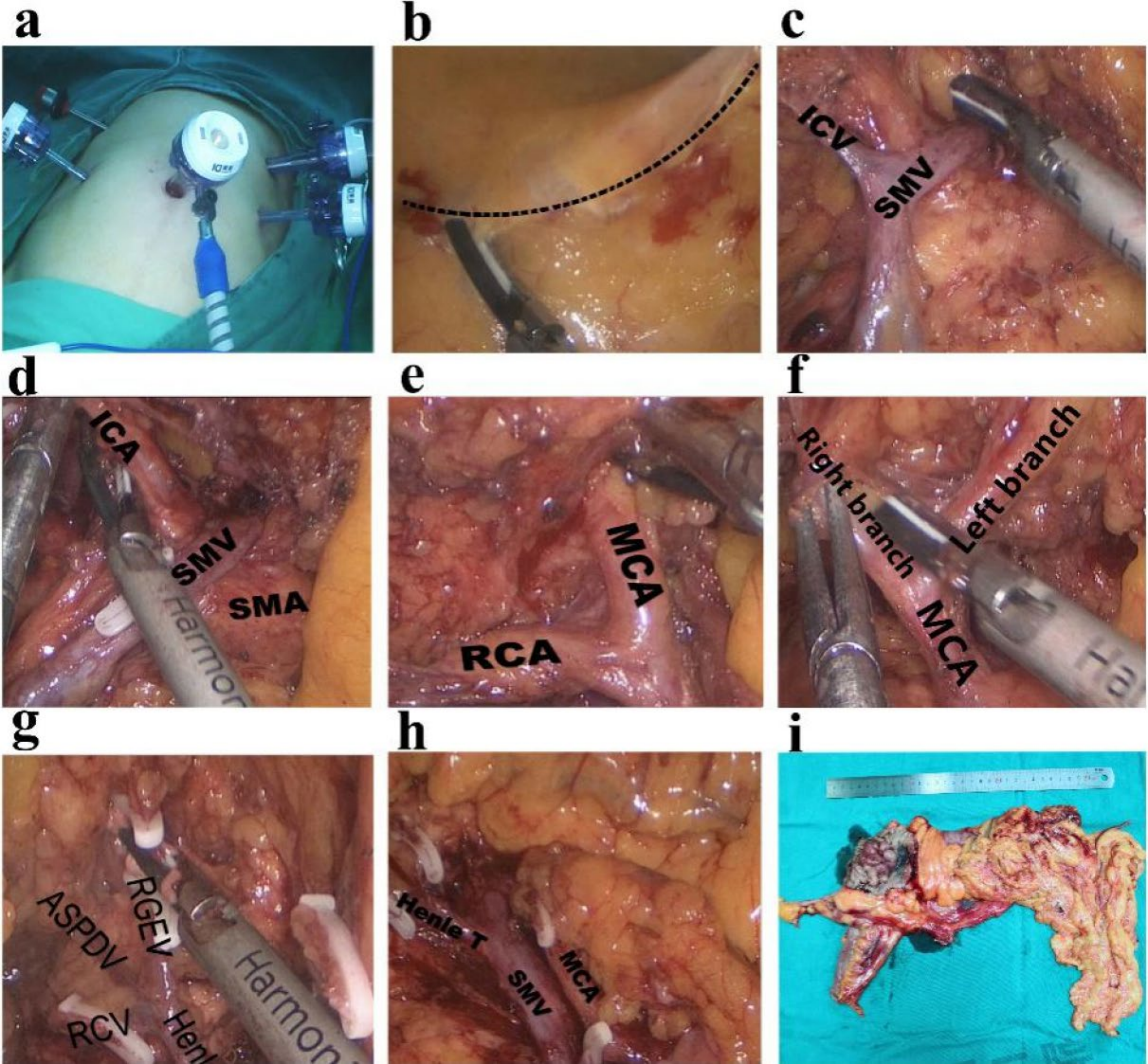
Surgical procedures for right hemicolon cancer: (**a**) trocar location, (**b**) opening of mesocolon above ileocolic vessels, (**c**) lymph node dissection at root of superior mesenteric vein (SMV) where the ileocolic vein (ICV) enters, (**d**) lymph node dissection at root of superior mesenteric artery (SMA) where the ileocolic artery (ICA) enters, (**e**) separation along right colic artery (RCA) and middle colic artery (MCA), (**f**) dissection of lymph nodes between left and right branches of MCA, (**g**) and (**h**) revealing anterior superior pancreaticoduodenal vein (ASPDV), right gastroepiploic vein (RGEV) and right colic artery (RCV), and dissected peripheral lymph nodes of Henle trunk (Henle T), and (**i**) excised right colon specimen.

**Figure 4:**
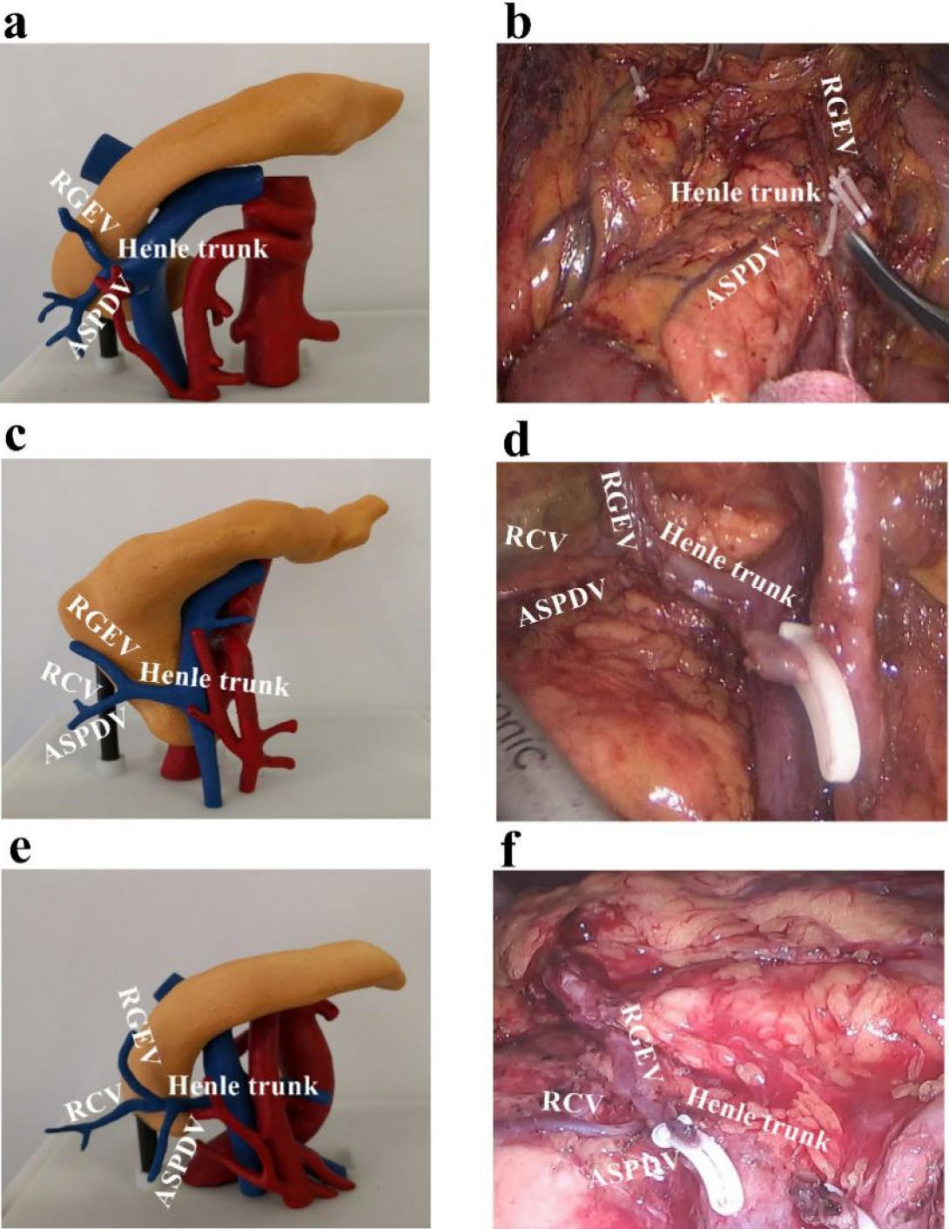
Anatomy and variation of superior mesenteric vessels: (**a**) 3D-printed model showing anterior superior pancreaticoduodenal vein (ASPDV) and right gastroepiploic vein (RGEV) converging into Henle trunk; (**b**) discovery during surgery, which is similar to 3D-printed model shown in (**a**); (**c**) 3D-printed model showing right colic vein (RCV) converging into ASPDV and then forming Henle trunk with RGEV; (**d**) discovery during surgery, which is similar to 3D-printed model shown in (**c**); (**e**) 3D-printed model showing RCV, ASPDV, and RGEV converging together into Henle trunk; and (**f**) discovery during surgery, which is similar to 3D-printed model shown in (**e**).

The completely medial approach was performed as described by Feng et al.^29,30^. In summary, the surgical approach was performed under general anaesthesia with tracheal intubation. The dissection started at the ileocolic vessel and proceeded along the superior mesenteric vein to enter the transverse retrocolic space in a bottom-to-top fashion. Then, the middle colon vessels, Henle trunk, and pancreatic lower edge were dissected. The operative procedures are shown in Fig. 3. The anatomy and variation of the superior mesenteric vessels in surgery are shown in Fig. 4, and the incidences of branches of the vessels are listed in Table 5. Surgery for right colon cancer can be considered a type of vascular surgery, since it is a surgery guided by blood vessels, and dissection is carried out along the blood vessels. In this study, short-term outcomes such as duration of surgery, bleeding volume, number of lymph node dissections, postoperative complications, postoperative flatus recovery time, duration of hospitalization, and medical expenses were collected.

**Table 5:** Incidences of branches of superior mesenteric vessels.

Variables	3D printing group	3D image group	Control group
ICA	20 (100%)	19 (100%)	22 (100%)
ICV	20 (100%)	19 (100%)	22 (100%)
RCA	9 (45.0%)	10 (52.6%)	12 (54.5%)
RCV	8 (40.0%)	8 (42.1%)	9 (40.9%)
MCA	20 (100%)	19 (100%)	22 (100%)
MCV	16 (80.0%)	17 (89.5%)	17 (77.3%)
Henle trunk	15 (75.0%)	15 (78.9%)	16 (72.7%)
Bipodal	3	4	4
Tripodal	7	6	8
Tetrapodal	5	5	4

ICA, ileocolic artery; ICV, ileocolic vein; RCA, right colic artery; RCV, right colic vein; MCA, middle colic artery; MCV, middle colic vein.

### Satisfaction survey

The satisfaction of patients was investigated using a questionnaire administered within 1 week after hospital discharge. The questionnaire was designed according to the content of the 2015 Medical Work Satisfaction Questionnaire in Jiangsu Province, China (Supplemental Fig. 1). Two options (Yes or No) or 4 options (very satisfied, satisfied, dissatisfied, very dissatisfied) were designed for each question. In order to facilitate the statistics, very satisfied and satisfied were considered as Yes, while dissatisfaction and very dissatisfaction were considered as No. Also, items 6, 7, and 8 were used to evaluate effective communication. Two or more “Yes” responses for these 3 items represented effective communication; otherwise, it represented ineffective communication.

### Statistical methods

Statistical analyses were performed using SPSS version 15.0 (SPSS Institute, Chicago, IL). Comparisons between the short-term outcomes were performed by Fisher’s exact test, the Mann–Whitney *U* test, or the Kruskal–Wallis test, as appropriate. A chi-square test was performed for the patient satisfaction data. A *P* value of less than 0.05 was considered statistically significant. The sample size of this study was calculated using PASS 15 Power Analysis and Sample Size Software (NCSS, LLC, USA) with α = 0.05, and 1 − β = 0.9, and a sample size of 10 (n = 10) for the control group, a sample size of 12 (n = 12) for the 3D-printing group, and a sample size of 13 (n = 13) for the 3D-image group were obtained.

### Ethics approval and consent to participate

The study was approved by the Ethics Committee of the Affiliated Wuxi No. 2 People’s Hospital of Nanjing Medical University.

### Consent for publication

We have obtained consent to publish from the participants to report individual patient data.

## Supplementary information

Supplementary information

## Data Availability

All data generated or analyzed during this study are included in this published article.
