# Supplementary material for: Usefulness of three-dimensional printing of superior mesenteric vessels in right hemicolon cancer surgery
Source: Sci Rep. 2020 Jul 15;10:11660. doi: 10.1038/s41598-020-68578-y (PMC7363793; doi:10.1038/s41598-020-68578-y)
Supplement: Supplementary file 1 — Supplementary information [file 41598_2020_68578_MOESM1_ESM.pdf]

Usefulness of three-dimensional printing of superior mesenteric vessels in right hemicolon cancer surgery

Yigang Chen, Linjie Bian, Hong Zhou, Danping Wu, Jie Xu, Chen Gu, Xinqi Fan, Zhequn Liu, Junyi Zou, Jiazeng Xiaand Zekuan Xu

Questionnaire on satisfaction of inpatients in general surgery

Dear patient:

Hello! Thank you for your trust in our hospital. In order to understand the medical service situation of each department in our hospital, make our work continuously improved and more close to your needs, please tell us the service situation of the medical staff truthfully. Thank You for Your Cooperation.

I wish you a speedy recovery.

Time: MM DD YY

1. Are you satisfied with the medical service during the hospitalization?
Very satisfied ( ) Satisfied ( ) Dissatisfied ( ) Very dissatisfied ( )

2. Are you satisfied with the attitude of the attending doctor?
Very satisfied ( ) Satisfied ( ) Dissatisfied ( ) Very dissatisfied ( )

3. Are you satisfied with the medical technology of the doctors in this hospital?
Very satisfied ( ) Satisfied ( ) Dissatisfied ( ) Very dissatisfied ( )

4. During hospitalization, are you satisfied with the time of communication between doctors and patients?
Very satisfied ( ) Satisfied ( ) Dissatisfied ( ) Very dissatisfied ( )

5. During hospitalization, are you satisfied with the way the doctor communicates with you?
Very satisfied ( ) Satisfied ( ) Dissatisfied ( ) Very dissatisfied ( )

6. During hospitalization, did the doctor clearly explain the operation process and postoperative precautions to you?
Yes ( ) No ( )

7. During hospitalization, did the doctor clearly explain the benefits and risks of the operation to you?
Yes ( ) No ( )

8. Do you think the doctor's explanation is helpful for you to understand the condition?
Yes ( ) No ( )

9. Are you satisfied with the medical charges during the hospitalization?
Very satisfied ( ) Satisfied ( ) Dissatisfied ( ) Very dissatisfied ( )

10. During your stay in hospital, do you have convenient and accessibility access to medical services?
Yes ( ) No ( )
